# A Comprehensive Review of the Role of Biomarkers in Early Diagnosis of Parkinson’s Disease

**DOI:** 10.7759/cureus.54337

**Published:** 2024-02-16

**Authors:** Somdutta Das, Harshal Ramteke

**Affiliations:** 1 Medicine, Jawaharlal Nehru Medical College, Datta Meghe Institute of Higher Education and Research, Wardha, IND; 2 General Surgery, Jawaharlal Nehru Medical College, Datta Meghe Institute of Higher Education and Research, Wardha, IND

**Keywords:** genetic biomarkers, imaging biomarkers, biochemical biomarkers, clinical biomarkers, early diagnosis, parkinson’s disease

## Abstract

Parkinson’s disease (PD) is a complex neurological, degenerative clinical condition depicted by the advancing loss of dopaminergic neurons in the substantia nigra pars compacta, which manifests itself as a myriad of sensorimotor and non-motor signs in patients. The disease occurs due to the reduced levels of the neurotransmitter dopamine in the brain, which is primarily associated with functional characteristics regarding mobility and cognition. The basal ganglion is mainly involved in the generation of cognitive functions and therefore is the most significantly associated area in PD. Since the classical diagnosis and assessment of PD depends majorly on the appearance of motor characteristics, which only arise when ~60-80% of the dopamine neuronal cell death has already occurred, it is imperative we focus on identifying biomarkers that can help us assess and diagnose PD in the earlier stages of disease progression, thus providing a better prognosis for the patients. This review article will focus on the different biomarkers that are currently available and in use, divided under the headings of clinical, biological, imaging, and genetic biomarkers, and assess their specificity and sensitivity toward providing an early assessment of Parkinson's for the patients and the future of preclinical diagnostics using molecular biomarkers. PD affects over 1% of the population worldwide and only ranks second to Alzheimer's disease in the context of its incidence and consequent socioeconomic burden. While recent breakthroughs in biomarkers have dramatically improved patients' odds of survival and prognosis, it still remains primarily a symptomatic diagnostic tool. It is an area of research that requires to focus on creating more advanced approaches toward diagnosing PD early, involving clinical diagnostics, neuroimaging technology, and molecular biology collaborations to provide the highest degree of care and quality of life that a Parkinson's patient deserves.

## Introduction and background

Parkinson's disease (PD) is a multifactorial, gradually escalating, neurodegenerative disorder whose etiology remains largely unknown, which is majorly treated symptomatically with no apparent cure in sight. It is thought to specifically target and damage the dopamine-producing (“dopaminergic”) neurons in a unique area of the brain known as substantia nigra pars compacta [1]. The distinctive pathological features of the disease, along with the aforementioned, are also inclusive of Lewy bodies formation by the deposition of α-synuclein (SNCA) protein inclusions in the surviving nigral neurons [2]. Parkinson’s ranks only second behind Alzheimer's disease (AD) in terms of its prevalence, at present affecting more than four million people globally, with numbers projected to increase by 100% in the next few decades [3]. It has been reported to affect about one out of 800-1000 individuals above the age of 60 years and four out of 100 of those with age over 80 years, making age one of the major known risk factors of the disease [1,2,4,5].

Dopamine acts as a neurotransmitter molecule in the motor and pre-frontal cortex, being associated with mobility, cognition, and other activities; as a result of the death of dopaminergic cells in PD, its amount is severely reduced in the diseased brain. Motor symptoms of Parkinson’s include muscle rigidity, resting tremors, postural disabilities, and bradykinesia although non-motor symptoms, such as depression, anxiety, altered sleep cycle, lethargy, cognitive disorders, urogenital, gastrointestinal, and sexual dysfunctions, may also arise in some individuals [2,5,6].

The apparent symptoms of the disease usually only begin to manifest after the nigral dopamine levels have significantly dropped, with the onset of molecular and cellular pathways, the neuronal pathology of PD likely taking place years before the appearance of the motor symptoms, leading to an almost 60-80% progressive degeneration of the nigral dopaminergic neurons over a course of approximately five to 15 years. The lack of apparent motor manifestations in the preliminary stages of the disease might be possible due to the presence of “neuronal reserve” or active compensatory mechanism(s) such as collateral axonal sprouting from the unaffected dopaminergic neurons [7].

Clinically, the challenges of the disease are inclusive of the fact that clinicians are unable to provide a definitive diagnosis at the earlier stages of disease progression and a reduced certainty in predicting the progression of the disease. The under-satisfied demand to recognize reliable, sensitive, and specific biomarkers for early identification and intervention in the disease progression of Parkinson’s is attracting a lot of attention in recent neurocritical advances. Therefore, finding accurate biomarkers is crucial to distinguish PD from other conditions, for early diagnosis of PD, including prognostic and pre-symptomatic diagnosis, to provide better clinical care and management at the very onset of the disease [6]. At the same time, those reliable markers might also essentially additionally be applied to track the evolution of the condition and are thought to expect beneficial results from a healing intervention [8].

In PD, between the initiating injury to cells in vulnerable nuclei of the central nervous system, and the appearance of diagnostic symptoms, there is a valuable period of clinical latency. Thus, screening of at-risk individuals in the interval of time between the anticipated commencement of dopaminergic cell death and the onset of diagnostic symptoms could be an essential criterion for the building and implementation of successful neuroprotective interventional therapies. The build-up of aberrant, fibrillary, intraneuronal accumulations of misfolded α-synuclein protein, which are known as Lewy bodies (presence of Lewy pathology) along with the degeneration and degradation of the dopaminergic neurons in the nigral striatal pathway, constitutes one of the major hallmarks of Parkinson’s neuropathology [2,3].

Due to a lack of effective clinical and laboratory biomarkers as diagnostic modalities to reliably diagnose pre-motor Parkinson’s, it is notoriously difficult for clinicians to anticipate the evolution of PD in an institutional setting, even in a vulnerable population until the clinical symptoms begin to manifest. The incidence of misdiagnosis in the early stages of PD has been reportedly as high as 25% [5]. It is, therefore, even more critical, in view of the current clinical scenario, that we create and implement the use of sensitive and specific biomarkers for prognostic diagnosis of the disease.

## Review

Search methodology

Eligibility Criteria

The eligibility criteria included all review articles and original studies that discussed the potential use of biomarkers of all different categories as an effective diagnostic and prognostic tool for PD. Studies that discussed the mechanism of action and effects of these biomarkers in patients of PD were also included. Articles and studies that discussed the use of novel diagnostic modalities such as genetic designation for the disease or protein identification were excluded from being a part of the main review. Articles published before the year 2000 were excluded from the narrative review. There were no other exclusion criteria.

Literature Search Strategy

We undertook a thorough systematic search through the PubMed electronic database. The complete duration of publications researched included articles from the year 2009 to the year 2023, and the last search was conducted on August 14, 2023. An extensive literature search was conducted using the key terms, including "Parkinson's disease," "Clinical biomarkers," "Genetic biomarkers," "Radiographic biomarkers," and “mi-RNA as biomarker for Parkinson's disease." These were combined with adjuncts of "AND" as well as "OR" to review specific subtopics of the article.

Data Extraction

**Figure 1:**
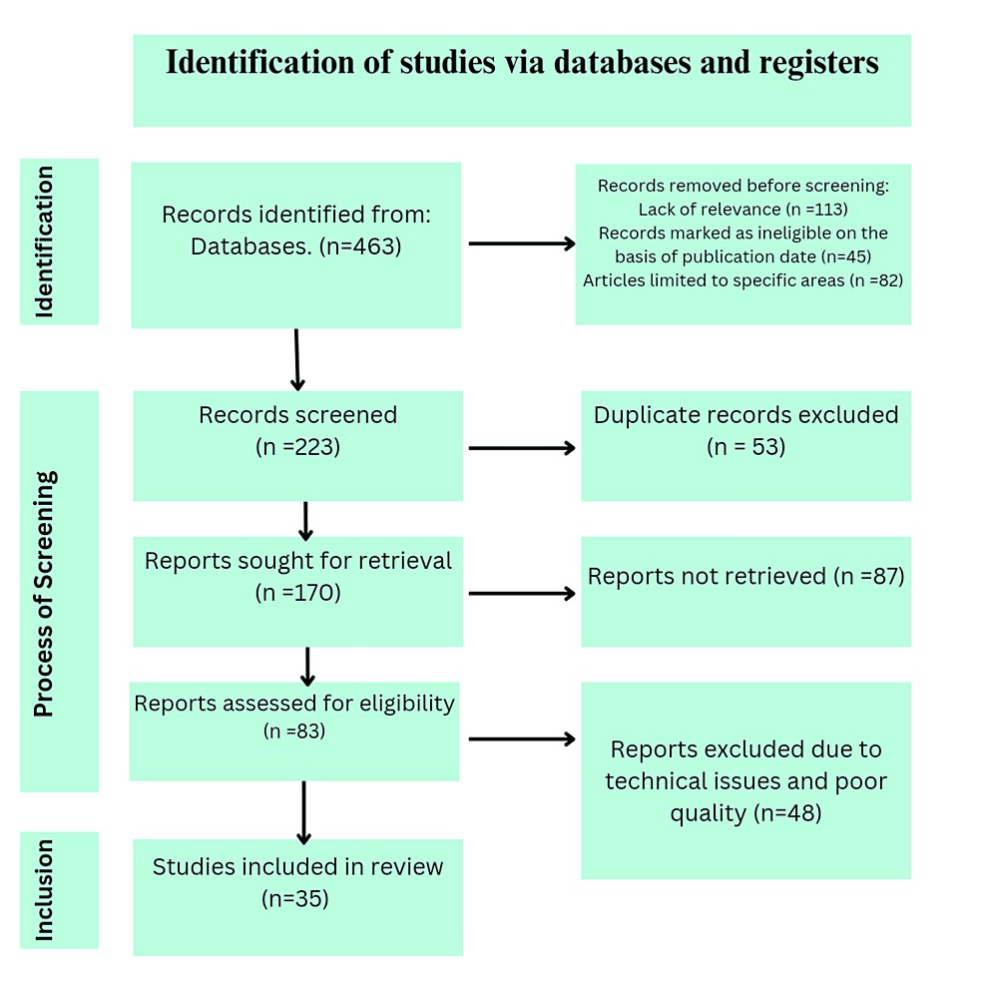
PRISMA flow diagram of search strategy. PRISMA, Preferred Reporting Items for Systematic Reviews and Meta-Analyses.

The abstracts of the research articles originated from the literature search were reviewed by authors. Those who met the selection criteria were studied and assessed further for their full texts. The search process and the number of studies included are demonstrated in a Preferred Reporting Items for Systematic Reviews and Meta-Analyses (PRISMA) flow diagram included below (Figure 1).

Biomarkers

In 1998, the National Institutes of Health Biomarkers Definitions Working Group provided the outline of biomarkers’ definition to be a functional feature that is assessed and evaluated as an objective indicator of regular biochemical machinery of the body, pathological progress, or pharmacologic outcomes of a healing intervention [1].

Therefore, biomarkers are essential to diagnose the presence of the condition early in the course of PD, while prevention and better prognosis are still achievable [9]. Pathophysiological assessments, bodily fluid or tissue examinations like that of cerebrospinal fluid and postmortem brain tissue, genetic or metabolic data, imaging measures, etc., are all, in general, considered biomarkers, classified under the methodological point of view [1,10]. Imaging, oxidative stress, neuroprotection, and inflammation are all included under some of the several biomarkers that might assist in providing early diagnosis of the disease to clinicians. Biomarkers are also utilized to collect information about the diagnosis and evolutionary progression of PD, either alone or in combination [11].

Biomarkers are clinically being utilized for various functions: (1) for attaining a favorable clinical outcome, e.g., to distinguish between PD and other neurological degenerative conditions like progressive supranuclear palsy (PSP), synucleinopathies, multiple system atrophy (MSA), dementia with Lewy bodies (DLB), cortico-basal degeneration (CBD), etc., especially to differentiate between idiopathic PD from other forms of parkinsonism [4,5]. Regardless of the invention of variations, they are between unique developers and none of them are sufficiently sensitive to aid in the identification and assessment of individual sufferers. Furthermore, generally, parallels are observed between affected patient corporations with a confirmed diagnosis, whilst we require biomarkers on the way to be beneficial in setting up a prognosis in a preclinical setting or in troublesome instances [12]. (2) Preclinical stage identification of any disease is incredibly pertinent for neurodegenerative disorders because biological alterations occur many years prior to the diagnosis in many such instances. While no disease-modifying intervention is presently accessible to us, as soon as we discover any significant one, biomarkers for the detection of early alterations could be extremely vital [13]. (3) A very essential characteristic feature of biomarkers is that they assist clinicians with expertise in underlying biochemical techniques leading to their differentiation, and consequently facilitating a successful intervention in that development. Genes, RNAs, microRNAs, proteins, peptides, and neurotransmitters, all can be included under molecules that can function as chemical biomarkers [14].

Clinical biomarkers

**Figure 2:**
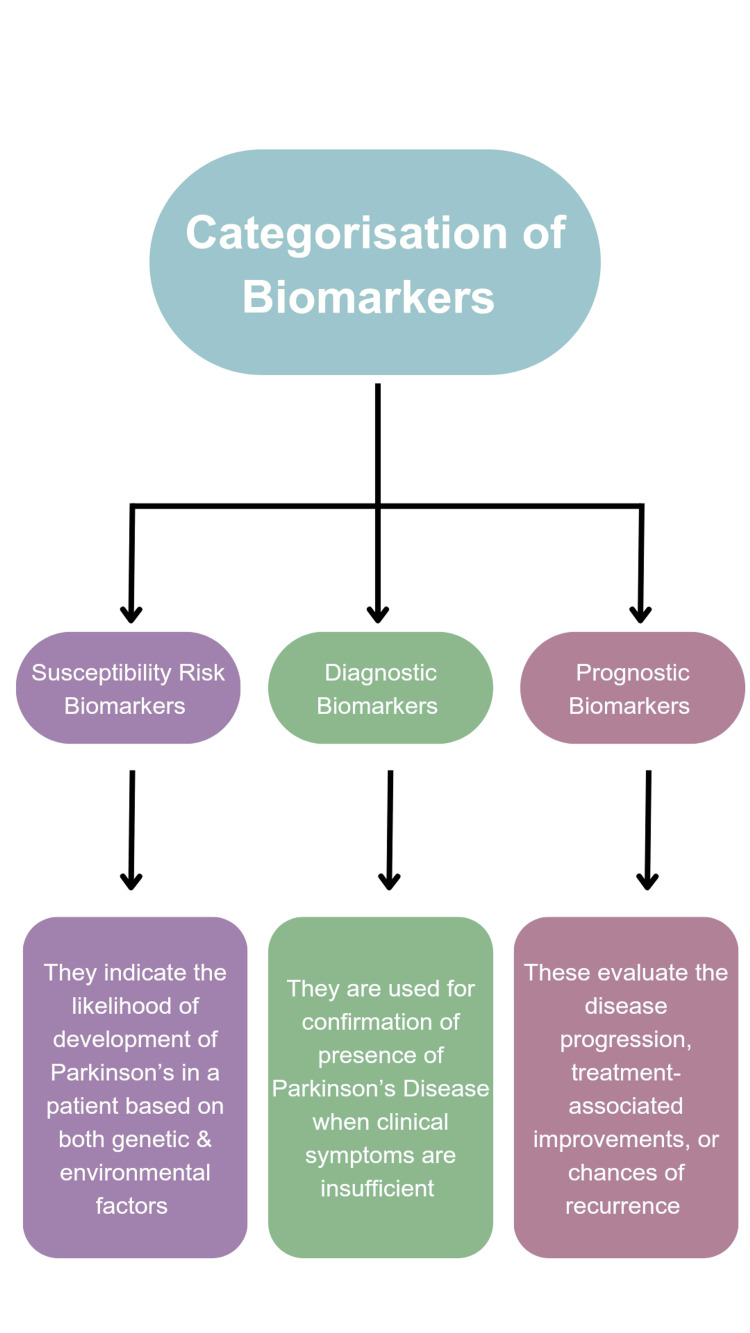
Classification of biomarkers based on their functional characteristics. Image credit: Somdutta Das [1,2,4,5].

PD biomarkers can be categorized into four significant types: clinical, imaging, biochemical, and genetic [13]. The oldest known and most significant clinical diagnostic and prognostic biomarkers of PD still remain the motor symptoms of bradykinesia accompanied by resting tremors and rigidity in muscles (Figure 2). It is the mechanical examination observation that resonates maximum with the diminution of dopaminergic neurons in the nigrostriatal circuit. A diagnostic measurement rating, such as the Unified Parkinson's Disease Rating Scale (UPDRS) or the modified edition of the UPDRS, the Movement Disorder Society-sponsored revision of the Unified Parkinson's Disease Rating Scale (MDS-UPDRS), is used to recurrently assess the accurate degree of motor impairment in patients [15].

Apart from motor manifestations, a few non-motor symptoms (NMS) appear unique for PD and also for other different synucleinopathies, which are neurological degenerative conditions characterized with the aid of aberrant fibrillary accumulations of α-synuclein protein in the CNS (like DLB and MSA) [16]. NMS may also emerge during the premonitory phase of PD, therefore being a potential candidate for the detection of prodromal PD. Early NMS signify the disintegration of neurons in the peripheral-nigral localities prior to the demise of nigral dopaminergic neurons and can be inclusive of hyposmia (olfactory disorder), rapid eye movement (REM) sleep behavior ailment, autonomic malfunction (including constipation), and melancholy [17,18]. We subsequently discuss some of the important NMS as diagnostic markers for PD.

Olfactory Dysfunction

Out of all the systems, the one system affected earliest in PD is the olfactory system, and therefore, hyposmia is considered an important NMS in PD. Studies have shown that approximately 75% of patients with Parkinson’s have an increased threshold for odor distinguishing, and about 90% of them ails from decreased odor identification but not all patients with PD suffer from olfactory symptoms with sensitivity ranging from 45% to 96% [1]. Therefore, it can be summarized that although hyposmia alone cannot be taken as a significant biomarker of PD, but when combined with other NMS and motor impairment assessments, it could provide us with a robust early diagnosis of PD [16].

REM Sleep Behavior Disorder

REM sleep behavior disorder (RBD) is a parasomnia affecting REM sleep and related to the prevalence of atypical nocturnal behavior and unusual motor or cognitive events as a result of a reduction of motor inhibition, which is a physiological phenomenon that is normally present in the course of REM sleep, i.e., the normal skeletal muscle atonia [15]. RBD is an early NMS that becomes apparent quite a while before motor symptoms do, and imaging studies in patients of RBD reveal a significant loss of symmetry in striatal dopaminergic uptake. Considering the number of patients with diagnosed RBD (polysomnography is the gold standard for diagnosis), a UPDRS score of >4 usually gives an indication of the presence of preclinical parkinsonism with an increased sensitivity and specificity about two years before clinical assessment, and a higher risk of parkinsonism is always correlated with a higher UPDRS score [15,19]. This can be considered to be a predisposition to the development of neurodegenerative disorders in 30-91% of the patients [20]. Thus, the presence of RBD as an NMS can become an important biomarker of early preclinical PD when evaluated with a combination of several biomarkers.

Constipation

Constipation is a significantly more sensitive early NMS biomarker that arises in PD, which affects up to 80% of patients with Parkinson’s [12]. It has been recommended that constipation springing up more than 10 years prior to the onset of cardinal motor symptoms, even up to 20 years prior is associated with a high prevalence, sustained risk of PD. Accumulation of α-synuclein and other neurodegenerative adjustments in the autonomic nervous system has been associated with the denervation of the myenteric plexus of the colonic sympathetic innervation [4]. These, along with oxidative stress and extended mucosal permeability have further been found to be in relation to colorectal transit time (CTT) that is usually extended in around 80% of untreated, de novo Parkinson’s patients, and may actually be a potentially evaluative biomarker for prodromal PD when studied with radio-opaque markers [21].

Proteomic markers in body fluids

Through the usage of proteomics, multiple attempts at research to identify the underlying pathology of disease development by identifying differential expressions of proteins and their adaptations as prospective biomarkers in biofluids from patients have been made recently [10]. The more recent proteomic techniques focused on the identification of a number of undifferentiated proteins in cerebrospinal fluid (CSF) (e.g., ceruloplasmin, chromogranin B, and apolipoprotein H), throw a promising light on early diagnostic measures for PD [22].

CSF is considered to be the ideal fluid candidate for detecting biomarkers of neurodegenerative disorders due to the absence of a barrier between the CSF and the brain, wherein α-synuclein species proteins, markers of amyloid and tau pathology, lysosomal enzymes, and neuro filamentous light chain in CSF present significant reflection of the disease pathology [8]. However, compared to blood and urine, collection of CSF is an invasive procedure, and in recent advancements, α-synuclein has been considered as a potentially important biomarker that crosses the blood-brain barrier and thus, can be easily detected in the plasma. The presence of misfolded aggregates of α-synuclein (Lewy pathology) is a hallmark in the pathogenesis of PD. It has been detected in its pathological (oligomeric, phosphorylated) form in CSF, blood, urine, saliva, etc. of patients and as some of these fluids are more readily accessible, it has developed into a potential investigative area for creating a biomarker assay to detect preclinical PD [8,23]. Other major potential biomarkers of CSF that have been studied include DJ-1 (Parkinsonism-associated deglycase) and several other variations of tau and neurofilament light chains, which might be useful in a differential diagnosis between various forms of parkinsonism. DJ-1 has also been studied in blood, where its levels are significantly higher than that of CSF, along with serum urate, levels of which have been found to be denominated in patients of PD than their normal, healthy counterparts for over two decades. Epidermal growth factor and apolipoprotein A1 are the other non-biased contenders of blood-based biomarkers for early diagnosis of PD [22].

Imaging biomarkers

Neuroimaging technologies have ascended to become mature biomarkers for evaluating nigrostriatal nerve degeneration and have gained popularity as non-invasive procedures to gain insight into not only the progression pattern of diagnosed PD but also as diagnostic in preclinical and prodromal stages [21,24].

Current imaging methodologies in use are transcranial sonography (TCS), utilized as an effective auxiliary assessment to directly visualize the structures in the midbrain and assess their echogenic features and characteristics qualitatively for the differential diagnosis of PD. Several studies on functional magnetic resonance imaging (MRI) techniques have provided evidential support of the nigrostriatal and nigrocortical connectivity as well as the criteria of nervous tissue or neural fiber integration that can signify RBD, which is considered to be a risk biomarker for early prodromal PD and therefore these MRI results can be supportive to a pre-clinical diagnosis of the disease before the onset of clinical Parkinson’s [3,20]. The dopamine transporter (DAT) imaging with single-photon emission computed tomography (SPECT) approach is being studied to have an overwhelming 98% sensitivity and specificity to scan for the functioning of presynaptic dopamine transporters and visualize the death of dopaminergic neurons in the nigrostriatal tract in PD [25]. Moreover, a DAT denomination at the groundline is concerned with the significant outcome of mobility and non-mobility significances of PD after 22 months [26].

While MRI biomarkers are upcoming as an advanced technological approach toward the clinical assessment of Parkinson’s, they still need to be cross-referenced with pathological findings to determine their reliability as a diagnostic modality and to assess their specificity and sensitivity to differentiate between Parkinson’s and other neurodegenerative conditions.

Genetic biomarkers

While no single gene cause of PD has been established so far, a genetic predisposition has been suspected in idiopathic PD, which has a complex, multifactorial etiology involving multiple influences, including lifestyle, genetics, and environmental factors. It has been studied that the chance of getting diagnosed with PD in individuals with a pre-existing familial history is three to four times higher than that of people who do not have any [16].

To date, as many as 20 genes have been linked with PD, eight genes out of which are thought to be concerned with an inheritance mode of autosomal-recessive type, out of which Parkin, DJ-1, and tensin homolog-induced putative kinase 1 (PINK1) are associated with the typical early-onset PD [27]. Some of the genes that are considered to be diagnostically important are α-synuclein (SNCA), PINK1, leucine-rich repeat kinase 2 (LRRK2), and β-glucocerebrosidase (GBA), and analysis for identifying mutations in them as diagnostics is still undergoing considerable study since they account for almost 2-3% of Parkinson’s patients [28]. Polymorphisms in genes such as LRRK2, S100B (S100 calcium-binding protein B), and NURR1 (nuclear receptor subfamily 4 group A member 2), along with neuroinflammation, were discovered to significantly elevate the risk for developing PD. Subsequently, these could be interpreted into biomarkers for diagnostic & prognostic purposes of PD, measuring the levels of the expression of their inflammatory proteins in CSF [29,30].

Global genome expression analysis with microarrays of DNA has been carried out on peripheral blood samples of individuals with Parkinson’s and compared with healthy control to conduct a comprehensive concept. Many studies have denoted mutations in the SNCA gene, which codes for alpha-synuclein (ASN), the main content of Lewy bodies, as the etiological factor of Parkinson’s [31]. However, in the future of preclinical diagnostic biomarkers, exploring more commonly detectable genetic biomarkers could be significant in identifying at-risk populations and better the evaluation of PD pathogenesis.

MicroRNA - the future

Whatever is now known by us regarding the potential mechanism of development of Parkinson’s disorder is simply the tip of the iceberg. It is quite tough to analyze the disorder, especially scattered, sporadic incidences at the genomic level, from the analysis of mutation of a single gene. The disbalance in genetic outcomes and phenotypical alterations is often caused by several diverse types of regulation techniques that function as mediators between genotypical and phenotypical expressions.

The microRNA (miRNA), which was proposed for the first time back in 1993, is considered one of those mediators. They are a collection of single-stranded, coding inefficient, tiny molecules that can, according to functional requirements, up or down-regulate the outcome of genes of their target by degeneration of mRNA or translation blockade [32]. Some down-regulatory miRNAs were assessed in human beings that lead to disorderly function of mitochondria, mitochondrial dynamics changes, oxidative stress, and the aggregation of SNCA, thereby resulting in the degeneration of neurons [33]. Almost all genes concerned with PD are controlled by these miRNAs, so they have been actively entwined into the pathophysiology of Parkinson’s. Particularly, α-synuclein has been seen to be targeted by two miRNAs, namely, miR-7 and miR-153. These two miRNAs reportedly demonstrate a synergistic function to downregulate the mRNA and levels of proteins of α-synuclein by binding to the 3′-untranslated region of α-synuclein [32]. Based on the evolution of PD and the exact stage of the disease, the miRNA expression can vary quite a bit, resulting in the heterogeneity of the miRNAs [34].

Since miRNAs that are circulating are theorized to be exactly organic to tissues, humongous, with increased stability, countable, and are up- or down-regulated for a few years, even decades prior to the onset of PD, a unique trajectory to employ miRNAs as non-invasive biomarkers to diagnose prodromal PD and assess the evolution of the pathology had been developed and theorized back in 2011 [35]. MiRNA profiling has the potential to be not only a cutting-edge technique for the early onset clinical finding of PD but also to embrace newer MiRNA-dependent therapeutics for better insight into improved prognosis of PD [32]. All the studies included in this review are summarized in the table below (Table 1).

**Table 1:** Summary of studies included in the review. PD, Parkinson's disease; CSF, cerebrospinal fluid; NADH, nicotinamide adenine dinucleotide hydrogen; EEG, electroencephalogram; MRI, magnetic resonance imaging; NMS, non-motor symptoms; iRBD, idiopathic rapid eye movement sleep behavior disorder; miRNA, micro ribonucleic acid; SNCA, synuclein alpha; LRRK2, leucine-rich repeat kinase 2; GBA1, glucosylceramidase beta 1; TLR, toll-like receptor; NF-κB, nuclear factor kappa B; NLRP3, nucleotide-binding domain, leucine-rich-containing family, pyrin domain-containing 3; NDD, neurodegenerative disorder; DaT, dopamine active transporter; SPECT, single-photon emission computed tomography.

Sr. No.	Authors	Year of publication	Country of origin	Summarization
1	Cova I et al. [1]	2018	Italy	Current research focuses on combining both clinical and non-clinical biomarkers to enhance diagnosis and advanced neuroprotective therapy to halt the progression of PD.
2	Chi J et al. [2]	2018	China	An integrated analysis of resources to identify significant genomic markers useful in developing targeted therapeutics for PD.
3	Chen-Plotkin AS et al. [3]	2018	USA	The recent development of a shared biofluid sample database led to focus biomarker efforts in PD to boost the therapeutic advent goals with global impact.
4	Erkkinen MG et al. [4]	2018	USA	A summary of the clinical aspects of the most commonly diagnosed neurodegenerative diseases along with a brief overview of diagnostic criteria, relevant imaging and laboratory findings, genetic basis, neuropathology, and management of the same.
5	He R et al. [5]	2018	China	The review discusses current advances in the development of PD biomarkers from various aspects and the diagnostic accuracy of a multimodal approach to biomarkers, which will facilitate the implementation of personalized therapeutic targets in patients.
6	Lotankar S et al. [6]	2017	India	This review comprises a discussion on various biomarkers available currently for PD and the recent advances in their development for early detection of the disease.
7	Miller DB et al. [7]	2015	USA	The advances in our understanding of the molecular mechanisms underlying PD may lead to novel biomarkers and new technologies for an advanced approach to biomarker development in the future.
8	Parnetti L et al. [8]	2019	Italy	Reliable prognostic markers such as a combination of CSF biomarkers, blood α-synuclein species, and neurofilament light chain could help in improving the prediction of response to treatment in PD.
9	Sharma S et al. [9]	2013	The Netherlands	This review describes coenzyme Q10, mitochondrial ubiquinone-NADH oxidoreductase, melatonin, α-synuclein index, Charnoly body, and metallothioneins as novel biomarkers to confirm PD for early and effective treatment of the disease.
10	Raghunathan R et al. [10]	2022	USA	Proteomics is capable of quantitation of high numbers of proteins from minimal sample volumes, hence this review focuses on recent proteomic studies and disease-related post-translational modifications in key proteins such as α-synuclein in PD, which may serve as biomarkers in recent times.
11	Fayyad M et al. [11]	2019	Qatar	The review highlights the novel techniques that have been employed for biomarker discovery and the evolving complexity in evaluating α-synuclein with regard to the considerable diversity of distinct conformers that exist in the biofluids under diseased states.
12	Waninger S et al. [12]	2020	USA	The review highlights the potential of EEG in effectively monitoring changes in neurophysiological oscillatory activity associated with PD and for effectively tracking disease progression.
13	Emamzadeh FN et al. [13]	2018	United Kingdom	This review encompasses biomolecules that might act as the biomarkers of PD, the risk factors (including genetics and non-genetic factors), and PD treatment interventional targets using genomic therapy.
14	Nalls MA et al. [14]	2015	USA	This study targets the development of a non-invasive, highly specific, and sensitive classification model for the diagnosis of PD, which could serve as a solid base for future disease prognosis and distinguishment.
15	Perlmutter JS [15]	2009	USA	A comprehensive review of rating scales for PD and a detailed guide on their implementation to derive the severity of the disease.
16	Ye H et al. [16]	2023	USA	Despite having hundreds of genetic loci, the data from experimental studies reveal identical neurobiological mechanisms of manifestations, leading to the development of successful treatment interventions in medicine for PD via targeted therapeutics.
17	Delenclos M et al. [17]	2016	USA	This review summarizes recent approaches to developing biomarkers as tools for the diagnosis and monitoring of PD along with novel strategies for their optimum utilization.
18	Ffytche DH et al. [18]	2017	United Kingdom	This study explores the spectrum of PD psychosis, the interrelation between neuropathology and functional MRI alterations, the role of medication in unmasking symptoms relating to worsening prognosis, and the future of clinical management and biomarker advent.
19	Arribarat G et al. [19]	2020	France	This study is aimed at providing an overview of recent developments in neuroimaging biomarkers for PD and their potential utilization in a clinical setting.
20	Le W et al. [20]	2017	China	A thorough discussion on the potential of recently discovered biomarkers for PD and their sensitivity and specificity in early diagnosis and risk evaluation of disease progression.
21	Ryman SG et al. [21]	2020	USA	The article evaluates the merits and limitations of utilizing MRI of the nigrostriatal system to detect broader dysfunction of NMS in PD and its effectiveness in monitoring the disease course.
22	da Costa AG et al. [22]	2011	Portugal	The study aims to review the validity of CSF proteins (α-synuclein and DJ-1) as effective biomarkers in the early detection of Parkinsonism and the novel proteomic techniques directed toward the detection of several undifferentiated proteins in CSF.
23	Hall S et al. [23]	2015	Sweden	The article presents evidence of a connection between raised α-synuclein at baseline and gradual worsening of motor symptoms and cognition over two years while also being a marker of more intense synaptic degeneration in PD.
24	Meles SK et al. [24]	2021	The Netherlands	Functional neuroimaging studies in iRBD provide an avenue in the development of newer therapeutic interventions as it represents an early prodromal stage of PD and therefore can be considered as a biomarker for early diagnosis.
25	Mitchell T et al. [25]	2021	USA	Neuroimaging biomarkers for several stages of PD are increasingly in demand to be used as potential outcome measures in clinical trials and are in utilization as multimodal combinations with routine assessment in clinical care settings.
26	Saeed U et al. [26]	2017	Canada	An overview of cardinal neuropathological features of neurodegenerative disorders is discussed in the article, followed by a discussion on imaging modalities as accurate and effective biomarkers for the evaluation of aforementioned syndromes.
27	Nies YH et al. [27]	2021	Malaysia	The article hosts a discussion on recent findings of PD-associated miRNAs' dysregulation and also updates on the potential effectiveness of miRNAs as novel diagnostic biomarkers and therapeutic advent for PD.
28	Polissidis A et al. [28]	2020	Greece	An overview of the advancements in therapeutic strategies developed for disease-modifying treatment and biomarkers in PD, with a focus on the most common genetic targets SNCA, LRRK2, and GBA1, and their applications in idiopathic PD.
29	Marogianni C et al. [29]	2020	Greece	The neuroprotective and immunomodulatory role of microglial activation in disease progression and the role of cytokines expressed in dopaminergic degeneration as biomarkers have been highlighted in this article.
30	Salemi M et al. [30]	2022	Italy	The aim was to identify miRNAs differentially expressed in PD in contrast to healthy control and if a specific pathway could be found to be associated with PD susceptibility along with their utility to be considered as potential diagnostic markers for PD.
31	Li Y et al. [31]	2021	China	This review provides a comprehensive summary of recent advances in the α-syn/TLRs/NF-κB/NLRP3 inflammasome axis of microglia as a useful advent for PD management by inhibiting microglial activation.
32	Mushtaq G et al. [32]	2016	USA	Increasing evidence is available suggestive of miRNAs' dysregulation in NDDs, thus circulating miRNAs within the blood may be identified as non-invasive diagnostic biomarkers that facilitate the early detection of diseases such as PD.
33	Weintraub D et al. [33]	2019	USA	The article explores the association between the neuropathophysiological basis for the appearance of psychiatric symptoms and exposure to certain dopaminergic drugs in PD.
34	Soto M et al. [34]	2022	Spain	Other than the clinical presence of iRBD or imaging biomarkers such as DaT SPECT, specific miRNAs also hold significant promise as progression biomarkers for patients with iRBD in predicting clinical outcomes of PD.
35	Li S et al. [35]	2022	China	The article summarizes the importance of microglial polarization in the progression of PD and the varied mechanisms by which miRNAs regulate microglial expression, thus highlighting their potential as therapeutic prospects.

## Conclusions

Parkinson’s is a complex, multifactorial neurodegenerative disorder wherein the cardinal symptoms leading to clinical diagnosis and therapeutic interventions appear quite late when the major loss of the striatal dopaminergic neurons has already occurred. Current advances in biomarkers are mainly focused on symptomatic diagnosis, neuroimaging to identify the progression of pathogenesis, and evaluation of levels of biomolecules in body fluids. Futuristic approaches, like genetic biomarkers and MiRNA-based assays, can significantly improve the prognosis of this disorder, providing a better management strategy to patients and enabling them to lead a robust life following a terminal diagnosis that is not only limited to a symptomatic cure.
